# Supplementary material for: Infection of army ant pupae by two new parasitoid mites (Mesostigmata: Uropodina)
Source: PeerJ. 2017 Oct 10;5:e3870. doi: 10.7717/peerj.3870 (PMC5639873; doi:10.7717/peerj.3870)
Supplement: Supplemental Information 1 — Figure S1. (A) Subcapitulum of M. derbyensis (F, OSAL 0106707). Peritrema of (B) M. derbyensis (OSAL 0119286) and (C) M. hilpertae (F, OSAL 0119286). (D) Tritosternum and (E) chelicera of M. hilpertae (F, OSAL0106708). Scale bars are 100 µm. Figure S2. Macrodinychus extremicus holotype. Figure S3. Museum voucher of Macrodinychus vietnamensis. Table S1. Definition of behavioral interactions. [file peerj-05-3870-s001.pdf]

Supplemental Material Overview

Infection of army ant pupae by parasitoid mites

Authors: Adrian Brückner, Hans Klompen, Andrew Iain Bruce, Rosli Hashim, and Christoph von Beeren

Content

Figure S1. (A) Subcapitulum of *M. derbyensis* (F, OSAL 0106707). Peritrema of (B) *M. derbyensis* (OSAL 0119286) and (C) *M. hilpertae* (F, OSAL 0119286). (D) Tritosternum and (E) chelicera of *M. hilpertae* (F, OSAL0106708). Scale bars are 100 µm.

Figure S2. *Macrodinychus extremicus* holotype.

Figure S3. Museum voucher of *Macrodinychus vietnamensis*.

Table S1. Definition of behavioral interactions.

The following files are downloadable at the Journal's webpage:

Supplemental video 1. Interactions between *Macrodinychus* mites and *L. distinguenda* host ants in laboratory settings.

Table S2. Behavioral assays and calculation of aggression index.

Figure S1. (A) Subcapitulum of *M. derbyensis* (F, OSAL 0106707). Peritrema of (B) *M. derbyensis* (OSAL 0119286) and (C) *M. hilpertae* (F, OSAL 0119286). (D) Tritosternum and (E) chelicera of *M. hilpertae* (F, OSAL0106708). Scale bars are 100 µm.

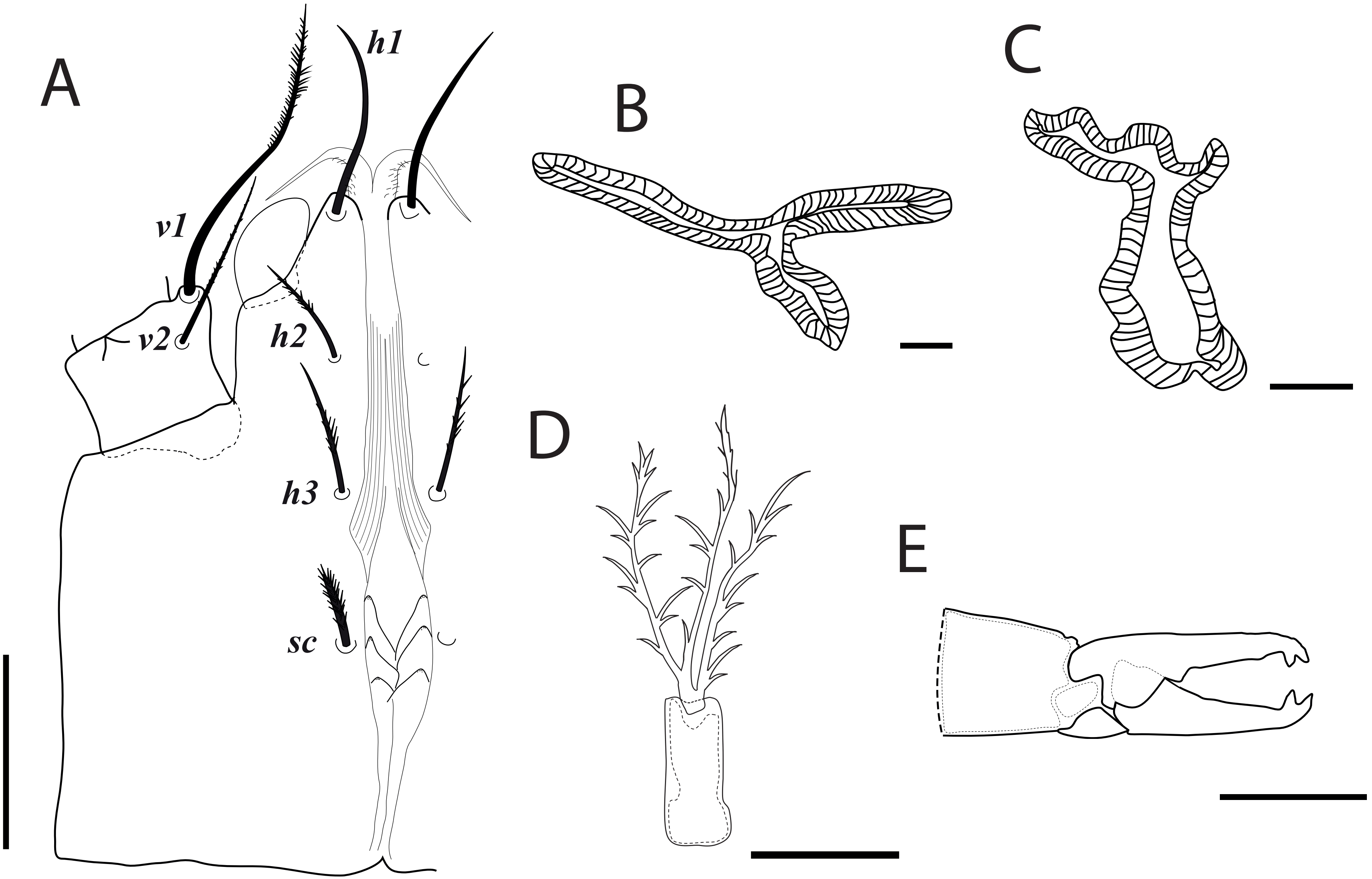

Figure S2. ***Macrodinychus extremicus*** **holotype.**
(A) Dorsal, (B) lateral, (C) and ventral view of *M. extremicus*.

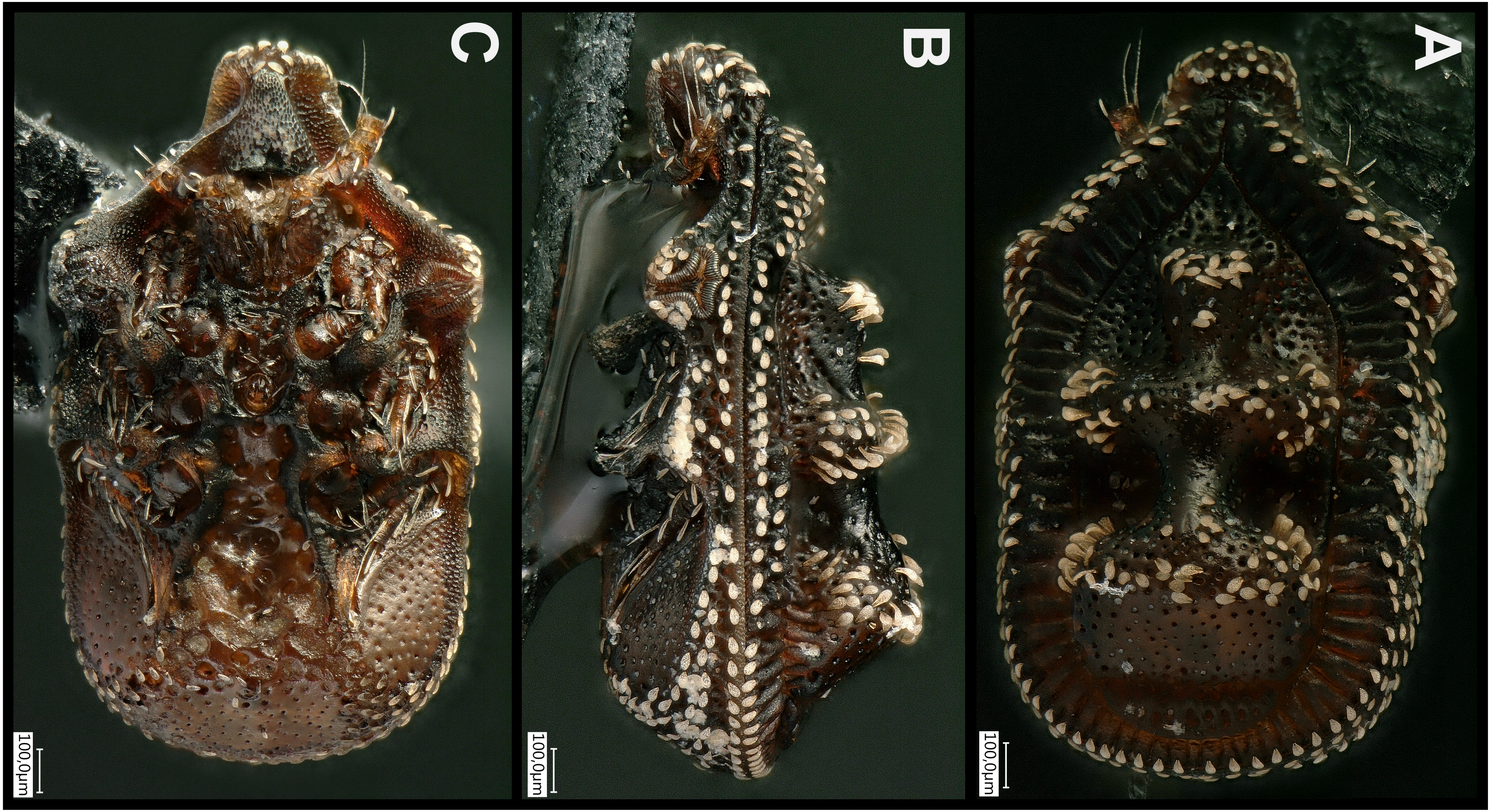

Figure S3. Museum voucher of *Macrodinychus vietnamensis*.
The holotype of *M. vietnamensis* is lost. These slide-mounted voucher slides, which were designated by Hirschmann, were used for comparative analysis. Vouchers are deposited at the Bavarian State Museum of Zoology. (A) Overview of slide-mounted material. (B, D, E) Ventral and (C) dorsal view (F) and close-up of sternal shield of slide 754. (G) Dorsal shield, (H) operculum, (I) part of dorsal shield and pygidial shield of slide 756.

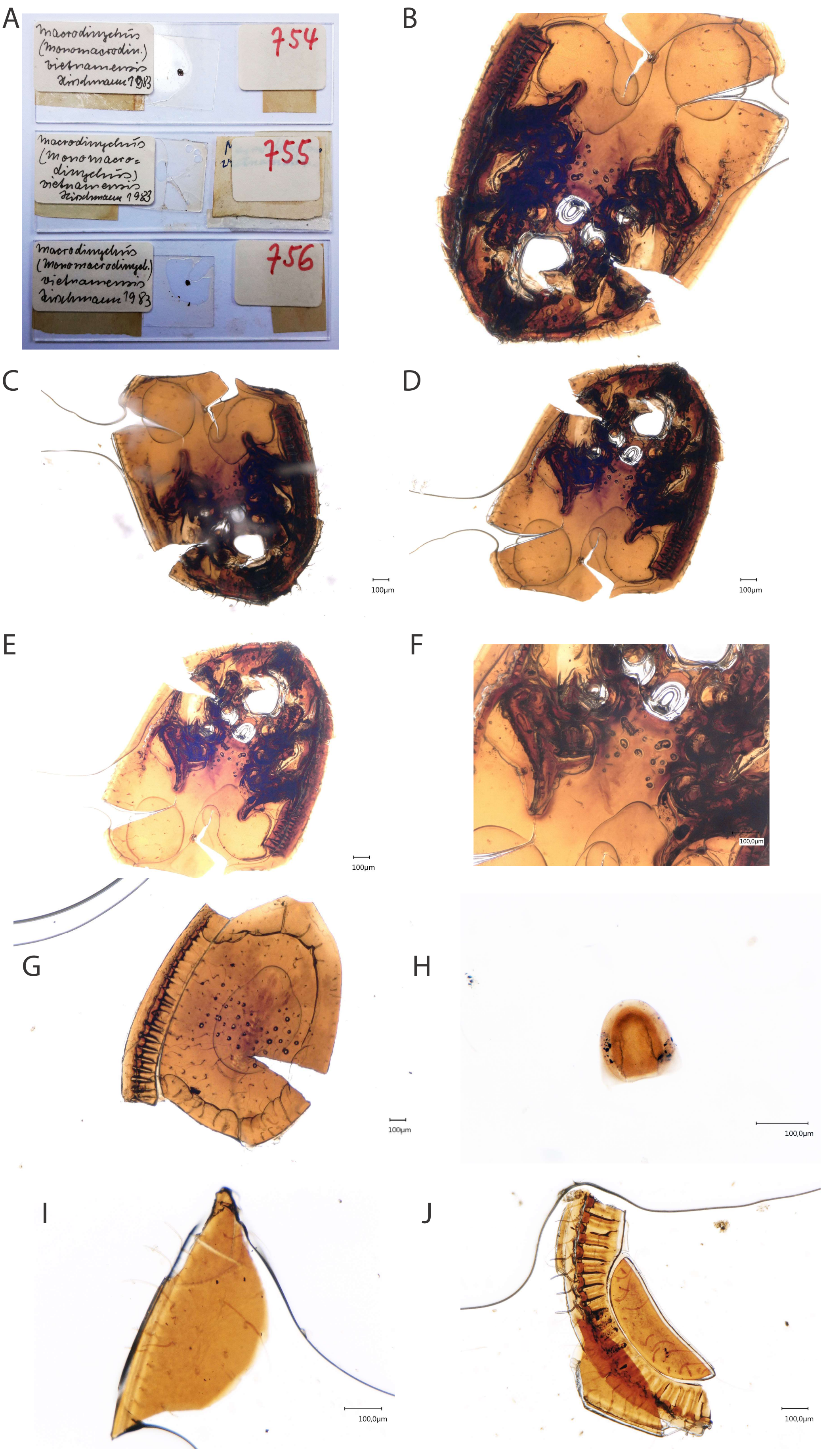

Table S1. Behavioral interactions between symbionts and ants. Each new contact between a focal myrmecophile and an ant was counted until a total of approx. 50 interactions was reached. Adapted from von Beeren et al. 2011. The behavior 'antennated' and 'ignored' are similar in that both behaviors include antennal contact between the ant and its opponent. 'Ignore' can be interpreted as a peaceful or neutral behavior; the opponent has not been recognized as intruder/potential prey. Antennation is interpreted here as a kind of inspection behavior of the opponent. Subsequent behaviors following antennation are often aggression or grooming behavior.

**Behavior**	**Definition**
Ignored	An ant worker touches the myrmecophile once with its antennae and moves on without any sign of behavioral modification.
Groomed	An ant grooms the myrmecophile with its mouthparts. The myrmecophile remains in position.
Avoided	When an ant approaches, the myrmecophile avoids contact by quick escape.
Antennated	An ant touches a myrmecophile repeatedly with its antennae for longer than two seconds without displaying other behaviors.
Unnoticed	An ant comes into and perhaps stays in contact with a myrmecophile, but not with its antennae; the ant does not modify its behavior.
Attracted by foam	Only in snails. The ant is attracted to the foam produced by the snail. The ant licks the foam intensively without losing contact.
Appeased	Only in beetles. The beetle lifts up its abdominal tip, obviously distracting ant workers (most likely by the release of defense compounds from its tergal gland).
Carried	The ant picks up the myrmecophile with its mandible and carries it around.
Chased	An ant touches the myrmecophile with its antennae and quickly lunges in its direction.
Snapped	An ant touches the myrmecophile with its antennae and snaps with its mandibles into the direction of the myrmecophile.
Stung	An ant touches the myrmecophile with its antennae, lunges forward and bends its gaster in direction of the opponent. The attempt does not need to be successful.
